# Environmental and Health Hazards of Chromated Copper Arsenate-Treated Wood: A Review

**DOI:** 10.3390/ijerph18115518

**Published:** 2021-05-21

**Authors:** Simone Morais, Henrique M. A. C. Fonseca, Sónia M. R. Oliveira, Helena Oliveira, Vivek Kumar Gupta, Bechan Sharma, Maria de Lourdes Pereira

**Affiliations:** 1 REQUIMTE–LAQV, Instituto Superior de Engenharia do Porto, Instituto Politécnico do Porto, R. Dr. António Bernardino de Almeida 431, 4200-072 Porto, Portugal; sbm@isep.ipp.pt; 2 GeoBioTec & Department of Biology, University of Aveiro, 3810-193 Aveiro, Portugal; hfonseca@ua.pt; 3 CICECO—Aveiro Institute of Materials, University of Aveiro, 3810-193 Aveiro, Portugal; sonia.oliveira@ua.pt; 4 Hunter Medical Research Institute, New Lambton, NSW 2305, Australia; 5 CESAM & Department of Biology, University of Aveiro, 3810-193 Aveiro, Portugal; holiveira@ua.pt; 6 Department of Biochemistry, Faculty of Science, University of Allahabad, Allahabad 211002, India; vivekguptaau@gmail.com (V.K.G.); bechansharma@gmail.com (B.S.); 7 Department of Medical Sciences, University of Aveiro, 3810-193 Aveiro, Portugal

**Keywords:** chromated copper arsenate, CCA-treated wood, arsenic, chromium, copper

## Abstract

Copper chrome arsenate (CCA) water-borne solution used to be widely used to make timber highly resistant to pests and fungi, in particular, wood products designed for outdoor use. Nowadays, CCA is a restricted chemical product in most countries, since potential environmental and health risks were reported due to dermal contact with CCA residues from treated structures and the surrounding soil, as well as the contamination of soils. However, large quantities of CCA-treated timber are still in use in framings, outdoor playground equipment, landscaping, building poles, jetty piles, and fencing structures around the world, thus CCA remains a source of pollutants to the environment and of increasing toxic metal/metalloid exposure (mainly in children). International efforts have been dedicated to the treatment of materials impregnated with CCA, however not only does some reuse of CCA-treated timber still occur, but also existing structures are leaking the toxic compounds into the environment, with impacts on the environment and animal and human health. This study highlights CCA mechanisms and the documented consequences in vivo of its exposure, as well as the adverse environmental and health impacts.

## 1. Introduction

Oxides of hexavalent chromium (47.5%), copper (18.5%), and inorganic arsenic (34%) are mixed in water to prepare a preservative of wood, known as chromated copper arsenate (CCA) [1,2]. CCA is used to protect wood or wood products and timber from insects, pests, and microbes by layering its fine green coating around wood or wood products that are used for indoor or outdoor purposes. In CCA, chromium, a transition metal, has no wood preservative properties. It acts as an agent to fix chemicals or their complexes in the timbers or wood by their binding with polysaccharides, i.e., lignin and cellulose. It is a very slow reaction process as the fixation of CCA with wood takes several weeks. Copper, another transition element, is primarily responsible for protecting the wood against decay by the action of microorganisms, such as bacteria and fungi. Arsenic, a metalloid, exhibits insecticidal properties. Arsenic also provides timber resistance to weather conditions, along with increased adherence of paint over a longer period [3].

**Figure 1:**
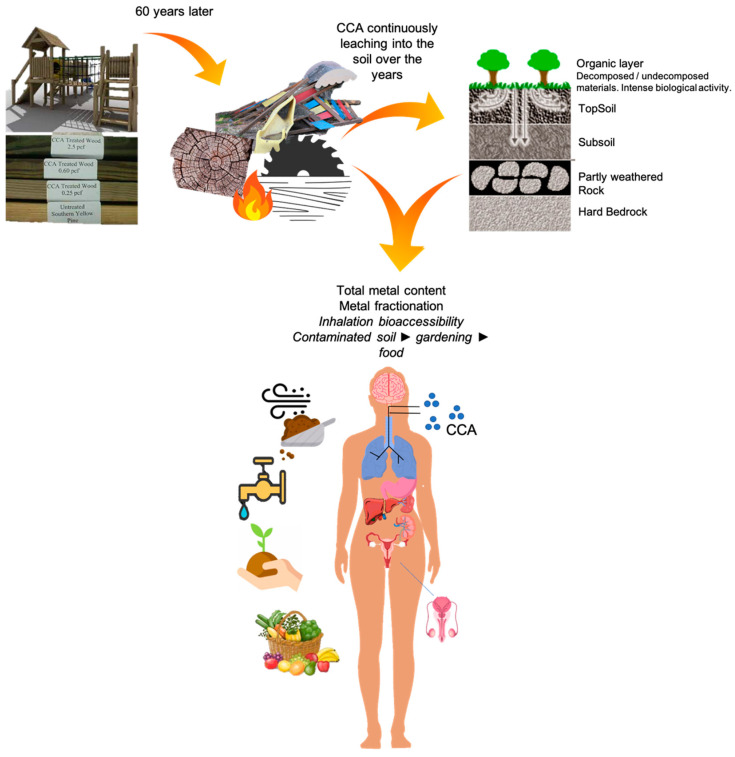
General overview of the potential environmental and human health impacts of chromated copper arsenate (CCA)-treated wood.

Wood products treated with CCA were found to have an adverse impact on the environment and human health, due to leaching and accumulation of these metals/metalloid, especially arsenic, from the wood into the environment (Figure 1). Decaying materials leach them into soils and waters, which may negatively impact food production or farming, and animal and human health. The affected tissues may include the brain, lungs, liver, stomach, spleen, kidneys, and reproductive organs [4,5,6,7,8,9,10]. The US EPA in 2003, therefore, agreed to reduce its use by adding only a minimum amount of arsenic to CCA [11]. In addition, CCA-impregnated wood products have been restricted to use either for burning or as equipment, such as decks, fences, landscaping features, patios, picnic tables, piling retaining structures, poles, on the playground and walkways [12]. However, such limitations for CCA use and application have not yet been set in some other countries, such as China. The toxic properties of many compounds of arsenic and chromium are known and extensively revised, but relatively limited information is available regarding the toxicology of CCA [4].

## 2. Systemic Effects of CCA-Research with Experimental Models

In the CCA-treated wood, arsenic may be present in association with chromium (III) or copper (II) in chemical forms such as chromium (III) arsenate (CrAsO_4_) and copper (II) arsenate (Cu_3_(AsO_4_)_2_), respectively, or as the quite stable clusters of chromium dimer-arsenic. It has been reported that the amount of arsenic, which leaches out to water and soil from the CCA-treated timbers at acidic pH (about 3.0), is sufficient to undergo bioaccumulation [2]. Hence, it is also toxic to aquatic and soil-based organisms [1]. The exposure of workers to CCA-coated timber promotes arsenic accumulation via inhalation with potential health risks [13]. In addition, children playing in parks or playgrounds can be exposed to CCA-treated wood products [14,15,16]. The toxic effects of CCA have been reported to be more severe than those of its individual constituents [4]. Our group investigated the effects of CCA and its compounds per se, using mice as models, and both hepatic and renal changes have been reported, with the former being much more heavily affected [5,6,7,8]. Carcinogenic effects promoted by co-exposure to Cr and As were observed in in vitro studies with normal human lung (BEAS-2B) and carcinoma (A549) [9]. More recently, N. Takahashi et al. [10] reported on the toxic effects of As and/or Cr on the hematopoietic, gastrointestinal, hepatic, and renal systems of Wistar Hannover rats exposed to 40 and 80 mg/kg/day.

### 2.1. Arsenic Toxicity

Arsenic exists in three different oxidation states; as trivalent arsenite (As (III)), pentavalent arsenate (As (V)), and elemental arsenic (As). Arsenite is 10 times more toxic than arsenate [17]. Other forms of arsenic include organic arsenic and arsine gas. This last form and inorganic arsenic are highly toxic. Routes of arsenic exposure include ingestion through food, inhalation, and absorption through the skin. Inorganic arsenic at 0.6 mg/kg acts as its lethal dose.

Chronic toxicity of arsenic has been studied in many animal body systems; some of the health effects are specific to the exposure, whereas most of the effects are systemic in nature. As indicated in reports from global health authorities and WHO [18,19], arsenic exposure causes damage to the mucous membranes, peripheral and central nervous systems, neuronal network, and hearing capacity. In addition, arsenic poisoning has been associated with the suppression of the immune system, as well as with increased fetal mortality in rats [20]. Children face particular health risks from exposure to arsenic [21,22,23].

#### 2.1.1. Arsenic as Carcinogen

According to the EPA Carcinogen Assessment Group, inorganic arsenic has been classified as a carcinogen belonging to Group A [24]. The exposure to arsenic through inhalation, ingestion and dermal contact may increase the probability of the occurrence of cancer in the bladder, kidney, liver, lungs, and skin [25,26,27,28]. After absorption, inorganic arsenic quickly reaches erythrocytes to bind with hemoglobin being transported to various organs within the body. Arsenic, in its methylated forms, (monomethylarsonic and dimethylarsinic acids) has a reduced level of toxicity [29]. Arsenic is shown to modulate the DNA binding abilities of certain factors, namely activator protein 1 (AP-1), transcription factor (NF-𝜅B), and tumor suppression protein (p53), which is responsible for the regulation of cell cycle. Valko et al. [30] have demonstrated that arsenic can activate c-Jun N-terminal kinases (JNKs) and extracellular signal regulated protein kinases (ERKs), which are responsible for promoting apoptosis and carcinogenesis, respectively [31,32]. In addition, a tumor-promoting transcription factor, AP-1, has been found to be activated by arsenic through MAPK and PKC [33,34,35]. Furthermore, Sun et al. [36] have revealed that arsenic may cause activation of the expression of an oncogene, specifically, a mineral dust-induced gene (mdig), through the activation of JNK and signal transducer and activator of transcription 3 (STAT3).

#### 2.1.2. Arsenic as an Oxidative Stress Agent

Arsenic as a constituent of CCA has been reported to induce a large amount of production of oxidative species in exposed animals, causing a serious imbalance in the redox systems [3]. The oxidative stress is a state of excess production of free radicals and increased degradation of antioxidant factors in an organism. Arsenic exposure has been shown to cause a significant reduction in the activities of redox-active flavoproteins such as viz. catalase (CAT), glutathione peroxidase (GPx), heme oxygenase-1 (HO-1), and superoxide dismutase (SOD), as well as in non-enzymatic peptides, such as glutathione (GSH), a sulfhydryl group containing tripeptide, also known as γ-l-glutamyl-l-cysteinyl-glycine [8,37]. GSH is capable of reducing As (V) into As (III). As a natural reducing agent and potential antioxidant, GSH effectively protects animal tissues from the toxic effects of arsenic-induced oxidative stress.

Arsenic is reported to cause damage to living cells and the DNA contained in it through enhanced lipid peroxidation and excessive production of oxidative species, such as NO and induction of the activity of poly (ADP-ribose) polymerase (PARP) [38,39]. Arsenic-induced oxidative stress has been found to be associated with damage to pancreatic islets and the occurrence of diabetes in the concerned individual [40,41]. Arsenic is reported to enter the central nervous system (CNS) and induce neurotoxicity by undergoing a process of biomethylation in the brain [30]. The acute lethal oral dose of arsenic is 1–2.5 mg/kg [42]. Arsenic poisoning results in the appearance of certain clinical features, such as anemia, weakness, abdominal pain, gastrointestinal troubles, diarrhea, vomiting, skin diseases, hypertension, encephalopathy, behavior changes, and malignancies in almost all the body organs [43].

### 2.2. Chromium Toxicity

The compounds of trivalent chromium display two to three times less toxicity compared to hexavalent chromium [44], which is present in CCA and has carcinogenic potential in humans [45,46]. An acute toxicity bioassay of the hexavalent chromium has been carried out both for the microorganisms and the aquatic invertebrates and the LD50 have been determined; the values being in the range of 50 µg/kg and 5 mg/kg for microorganisms for 48 h. The 48 h LC50 values of hexavalent chromium for aquatic invertebrates and fish were found to be in the range of 66 µg/L to 64 mg/L and 17.6 to 249 mg/L for different fish species.

Human exposure to hexavalent chromium may occur through inhalation, ingestion and absorption by dermal contact [47]. Chronic human exposure to hexavalent chromium results in irritation and rashes on skin, and corrosion and irritation in the airways of the respiratory system, causing damage to the mucous membranes and development of lung cancer [48,49]. The results of the studies on chromium (VI) released from industrial emissions have shown it to be highly toxic in nature due to its very strong oxidative properties and smooth membrane permeability. Chromium (VI) has been found to show nephrotoxicity and hepatotoxicity. In addition, the teratogenic effects of chromium (VI) have also been detected in exposed animals [50,51].

### 2.3. Copper Toxicity

The acute copper toxicity bioassays conducted with aquatic invertebrates have indicated LC50 values for 48 h in the range of 5 µg/L to >10 mg/L. It was observed that the aquatic invertebrates containing hard exoskeletons, for example mollusks, and the marine arthropods were more tolerant to the toxic effects of copper than the aquatic organisms without shells. Copper has been found to exert toxicity to fish and several other marine vertebrates.

Copper is a known essential trace element for normal human metabolic activities. However, its high concentrations have been found to pose toxic effects on both humans and other mammalian systems [48]. Anomalies in copper metabolism or mutations on genes-related copper metabolism induce Wilson disease, where mutations in the ATP7B gene take place, giving rise to increased levels of copper and consecutive toxicity [52,53]. If left untreated, this disease develops into liver failure or severe neurological deficiency and death.

## 3. Bioavailability and Bioaccessibility of CCA Compounds

There are fewer studies on the bioavailability and bioaccessibility of CCA-treated wood contaminants when compared to those that focus on the contamination of soils by arsenic, chromium, or copper (Table 1). The term bioavailability in the literature assumes somewhat different meanings depending on the context. For example, from a pharmacological point of view, it is generally considered to be the rate and extent at which moieties are absorbed and become available in cells and tissues. From a nutritional point of view, it refers to the intake fraction that is stored or made available in physiological functions. Bioaccessibility, on the other hand, is described as the amount of a compound released from its inactive form, in the gastrointestinal tract, by means of digestive transformation, thus becoming available for absorption and assimilation by cells [54]. It is generally assumed that bioavailability is the amount of chemical absorbed by the organism. It depends on factors, such as the species of the product in question, the time and type of matrix in which it is present, and the way in which the exposure occurs. Ingestion and direct contact through the skin of the hands are the predominant forms of human exposure, especially in children under six years of age. If ingested, bioavailability is influenced by the dissolution of chemicals in gastrointestinal fluids and their absorption via the gastrointestinal tract into the bloodstream. Biomonitoring based on several biological fluids (urine and saliva) was used to assess timber workers’ and children’s exposure to the most hazardous CCA components, in particular chromium and arsenic [13,14,15,16]. Occupational data clearly demonstrated elevated urinary concentrations of the selected biomarkers, namely inorganic arsenic and chromium, in CCA-exposed plant laborers when compared with the levels of non-exposed subjects [13]. Concerning child biomonitoring studies, evidence is inconclusive, with studies based on urine and saliva suggesting that CCA playgrounds only slightly contribute to the total arsenic exposure of children [14,15,16]. The impact of consumption of contaminated food seems to be the prevalent contributor of arsenic burden in children, in contrast to the case of occupationally exposed individuals. Still, all studies detected a direct relationship between arsenic levels on the children’s hands after playing on CCA-treated playgrounds [14,15,16,55]. Moreover, authors are unanimous in stating that mitigation strategies are needed to minimize total arsenic exposure. Recently, the levels of As on the surface soils around some play structures installed 16 and 26 years previously were evaluated by high-performance liquid chromatography–inductively coupled plasma mass spectrometry (HPLC–ICPMS) and in vitro SBRC-gastric assay, and the authors demonstrated that ≤29% of As was bioaccessible, underlining concerns about potential health risks for children [56]. CCA-wood enclosures for zoological gardens also represent a risk for captive animals, due to the leaching of As into the soil [57].

The bioavailability and bioaccessibility of CCA have been studied in organisms ranging from soil micro and macro-organisms to humans (Table 1). This diversity of data sources encompasses the need to fill gaps in knowledge about the capacity of the cell, the organism and the environment, in order to deal with the negative impact of CCA. Ultimately, and in a more anthropocentric perspective, this diversity of studies provides important information for understanding the mechanisms that determine the impact of these compounds on human health.

Table 1 allows a quick overview that summarizes the research in a variety of different sites and conditions, using a combination of techniques, such as specific bacterial bioreporter assays, in vitro solubility bioaccessibility research consortium (SBRC)-gastric assays, simulating gastrointestinal tract conditions and, or in combination with, spectrometric methods (ICP-MS, inductively couple plasma-mass spectrometry; ICP-OES, inductively coupled plasma-optical emission spectrometry) and high-performance liquid chromatography. For further insight into each specific study, the reader is directed to the relevant publication (Table 1, last column). Overall, the reported data demonstrate dependence on the variability of different parameters, such as type of organism, medium and route of exposure, source fraction and concentration of the contaminating source, as well as leaching, immobilization and intake parameters. Several studies also explore the effect of oxidation of dissolved ferrous iron on reducing the bioavailability of As, through the adsorption and precipitation on iron oxides (e.g., [58]). For Cu-bioavailability, the use of biochar has been explored either alone (e.g., [59]) or in conjunction with dissolved ferrous iron when anionic trace element contaminants are also present (e.g., [58,60]). Frick et al. [60] demonstrated that the bioavailability/bioaccessibility values are particularly influenced by physicochemical factors that govern the solid phase distribution of As, Cr and Cu in CCA-contaminated sites. The calculated risks for environmental and human health are the result of the combined impact of natural processes and anthropogenic inputs. The former includes soil acidification by leaching rainwater, and the chemistry of organic matter and plant exudates, in combination with the ageing processes of soil and wooden structures. Among anthropogenic inputs, we may list the targeted remediation approaches and land use that can alter the solid phase distribution and availability of contaminants. These all have an impact on the calculated risks for environmental and human health [59].

**Table 1:** Summary of bioavailability/bioaccessibility data of contaminants from CCA-treated wood sites.

Organism	Exposure Medium	Exposure Route	Fraction of Source	Total Concentration	Total Background Concentration	Bioavailability Test	Bioavailability/Bioaccessibility	Ref.
**Whole-cell bacterial bioreporters**								
	Soil	As, *Escherichia coli* pJAMA arsR; Cu, *Pseudomonas* *fluorescens* DF57-Cu15	Soil-water extractable metalloid	Soil: As, 1364; Cr, 540; Cu, 1662. (mg /kg)	n/a	Bioreporter’s specific gene expression *; and analyzed by ICP-MS	(Bioavailable) As, 6.1 ± 0.8; Cr, na; Cu, 8.2 ± 0.6 (mg/kg) **	[58]
**Earthworm (*Eisenia andrei*)**								
	Humus soil layer	Earthworms ingestion	Soil from a 60 year-old Norway spruce (*Picea abies* L.) stand	Soil: As, 10.1 ± 5.5–2810 ± 921; Cr, 12.5 ±10.6–1480 ± 355; Cu 5.14 ± 5.3–642 ± 180 (mg/kg)	As, 6.12 ±1.2; Cr, 5.3 ± 1.7; Cu, 4.7 ± 1.1 (mg/kg)	Animal chemical digestion and analyzed by ICP-OES	(Bioavailable) As, 22–357.1; Cr, 0.9–40.8; Cu, 7.7–51.1 (mg/kg body eight) **	[61]
**Children**								
	Surface soil (<250 μm fraction)	Incidental ingestion	CCA-treated playground structures 16 and 26 yrs-old installation	Soil: As, 101.3–213.5 (mg/kg)	As, 4.6–6.6 (mg/kg)	In vitro SBRC-gastric assay	(Bioaccessible) As, 24.5–29.4% of total As (109–236 mg/kg) in the <250 μm fraction	[62]
**Plant (*Spinacia oleracea*)**								
	Artificial soils: sandy soil with 3.8% coir peat, 13.5% perlite and 82.7% sand; clay soil with mixing sandy soil with 10% bentonite	Spinach leaf and root	Irrigation with untreated leachate; water from submerged timber blocks; and irrigation with tap water (no As, Cr and Cu) on soil mixed with shredded timber (powder < 15 mm)	Soil: As, 5–176; Cr, 5–252; Cu, 5–127 (mg/kg)	n/a	Plant chemical digestion and analyzed by ICP-MS	(Bioavailable) Sandy soil (Leaf) As, 0.2–1.5; Cr, 0.5–2.9; Cu, 0.8–4.4; (Root) As, 20–62; Cr, 0.6–1.3; Cu, 2.3–10 Clay soil (Leaf) As, 0.3–0.9; Cr, 0.3–1.8; Cu, 1–4.24; (Root) As, 9–148; Cr, 0.7–61; Cu, 3.8–59 (mg/kg plant wet weight) **	[63]
**Whole-cell bacterial bioreporters children**								
	Soil	Cu-specific *Pseudomonas* *fluorescens* bioreporter	Soil-water extractable concentration: As, 0.17–18.3; Cr, 0.02–0.78; Cu, 0.11–5.99 (mg/kg)	Soil: As, 32.4–2839; Cr, 26.1–1819; Cu, 17.2–2205 (mg/kg)	n/a	Bioreporter’s specific gene expression *	(Bioavailable) As, na; Cr, na; Cu, 0.04–3.52 mg/kg	[64]
**Whole-cell bacterial bioreporters children**								
	Soil and wood treated staircases/railings	Hand-to-mouth incidental ingestion	Soil and surface wipe (50 cm^2^) (construction years from 1978–1998)	Soil: As, 1.2–66.6 mg/kg. Surface wipes (dislodgeable As): 5.4–86.1 μg/100 cm^2^	Soil: As, 1.2–3.1 mg/kg. Surface wipes: As, <0.2 μg/100 cm^2^	Soil: In vitro SBRC-gastric assay; Surface wipes: analyzed by ICP-MS	(Bioaccessible) Soil: As, 1.2–25.2 mg/kg (17–84%). Hand loadings: As, 15–23.8 μg/100 cm^2^	[65]
**Human**								
	Soil	Incidental ingestion	Soil-water extractable metalloid	Soil: As, 170 ± 35 mg/kg	n/a	In vitro gastrointestinal bioaccessibility	(Bioaccessible) As, 30.5 ± 3.6% (17 ± 0.4–46 ± 1.1%)	[66]
**Earthworm (*Eisenia fetida*)**								
	Soil	Earthworms Ingestion	CCA-treated wood–water leachates	Soil: As, 13–169; Cr, 12–151; Cu, 10–216 (mg/kg). Wood leachate As, 325 ± 4; Cr, 291 ± 3.4; Cu, 248 ± 4.2 (mg/L)	Untreated wood leachate: As, 0.5 ± 0.7; Cr, 0.35 ± 0.5; Cu, 0.55 ± 0.8 (mg/L)	Earthworm growth and reproduction test; and analyzed by ICP-OES	(Bioaccessible) Ranged from negligible to As, 80; Cr, 89; Cu, 90 (mg/kg)	[67]
**Children**								
	Soil	Incidental ingestion	Soil immediately adjacent CCA-treated utility poles after 18 months of service	Soil: As, 37.4 ± 2.5–251 ± 12 mg/kg	n/a	In vitro astrointestinal method	(Bioaccessible) As, 25.0 ± 2.7–66.3 ± 2.3 % (mean value 40.7 ± 14.9%)	[68]
**Human / mix of plant species**								
	Soil	Phytotoxicity	Soil used from 1942 to 1968 for CCA wood impregnation	Soil: As, 5904 ± 194; Cr, 3829 + 161; Cu, 1509 ± 90 (mg/kg)	n/a	Physiologically based extraction test [69]; and analyzed by ICP-OES	(Bioaccessible) Soil: As, 219 ± 10; Cr, 26.1 **; Cu, 581 ± 30. Plant shoots: As, 78.6 ± 0.3; Cr, 7.4 ± 0.1; Cu, 48.5 ± 0.2 (mg/kg)	[60]
**Soil microbial community structure**								
	Soil	As, *E. coli* plasmid R733 recombinant with plasmid pTOO31	Soil metal contamination from 1950 to 1998	Soil: As,190–2500; Cr, 250–1900; Cu, 140–950 (mg/kg)	As, Cr and Cu were below 80 mg/kg	Bioreporter’s specific gene Expression *; and analyzed by ICP-MS	(Bioavailable) As, 0.25–10.3 mg/L (5.1–42.3% of total water-soluble As concentration)	[70]

n/a; not available. (mg/kg) when not stated otherwise, refers to milligram per kilogram dried weight. (*) Bioavailability defined as metalloid concentration capable of inducing bioreporter’s specific gene expression within an incubation period. (**) Approximate values retrieved from graph.

## 4. Removal of CCA

The concern with the disposal of wood residues treated with CCA has grown due to the risk of environmental contamination. Open burning of CCA-treated wood products has shown to emit 11–14% of the total arsenic content into the atmosphere (in contrast with chromium and copper, emissions of which contain less than 1% of the total), with the remaining arsenic in the residual ash. Moreover, the identified oxidation states of the CCA components in the particulate matter were As (III) and As (V), Cr (III), and Cu (I) and Cu (II), suggesting that open burning of CCA-treated wood may be the origin of the more toxic trivalent form of As in inhalable particulates. Acute and chronic arsenicism have been described due to burning of CCA-treated wood [71]). Consequently, the USA Consumer Product Safety Commission has recommended to not burn CCA-treated timber [72]. Thus, the release of CCA-treated wood components is an increasing environmental concern and strategies to remove copper, chromium, and arsenic from treated waste wood are required for economic and environmental purposes. By using effective methodologies to remove components, wood fibers can be recycled into composite products.

CCA-treated wood presents chromium in largest proportion, making it the main challenge in the extraction process, as chromium has the strongest affinity to wood lignin, so it is the most resistant to extraction [73].

Several approaches have been used to remove CCA components from treated wood; acid extraction with citric, acetic, formic, oxalic, nitric, or sulfuric acids are the most common approaches [73]. Acid extractions are usually combined with steam explosions, bacteria or fungi that can tolerate the high levels of metals present in CCA-treated wood. In fact, the most efficient strategies in removing CCA involve combined methods.

Claus and co-workers [67] tested the wash off of CCA from treated wood using oxalic acid extraction, steam explosion and bacterial fermentation with *Bacillus licheniformis* CC01. Steam explosion as a mean of opening the chemical structure of wood for releasing copper, chromium, and arsenic demonstrated low applicability [74]. One of most efficient methods is the chemical fiber modification with oxalic acid, which eliminates 62–89% of copper, chromium, and arsenic from CCA-treated wood scobs. In addition, these authors have also combined some microbial and mechanical methods to remove all components of CCA from treated wood [74]. The combination of steam explosion with further oxalic acid extraction or bacterial fermentation showed a lower release of components from treated wood, with values of 35% depletion in chromium, whilst values were almost null when using the oxalic acid extraction *per se*. In relation to the extraction with oxalic acid as a precursor to *Bacillus licheniformis* CC01 fermentation, high values of removal were obtained (90% copper (CuO), 80% chromium (CrO_3_), and 100% arsenic (As_2_O_5_)), which make it the most efficient treatment combination to clear relevant amounts of metals from CCA-chipped wood. In another study with CCA-treated wood wafers, Clausen and co-workers [75] found that 18 h exposure to oxalic acid provided a most favorable release of copper, chromium and arsenic from CCA-treated wood. The reductions of 78% copper, 97% chromium and 93% arsenic were obtained using 0.80% acid extraction combined with culture of *Bacillus licheniformis* CC01 [75].

The microbial conversion of CCA-treated wood has been also achieved by brown rot fungi of the genera Antrodia and Meruliporia, recognized for their tolerance to copper and generation of high levels of oxalic acid. The great generation of oxalic acid enhances the acidity of the substrate, promoting the solubility of chromium and arsenic; this can be applied as a possible method to commercial oxalic acid.

Other authors demonstrated a high decrease in copper, chromium and arsenic levels by brown rot fungi *Fomitopsis palustris*, *Coniophora puteana* and *Laetiporus sulphureus* [76], *Daedalea dickinsii* and *Polyporales* (unkown sp) [77]. High rates of removal of copper, chromium and arsenic (96%, 92% and 98%, respectively) were reached by *Fomitopsis palustris* [77].

Dos Santos and co-workers showed that the acid extraction of CCA components from *Eucalyptus sp*. and *Pinus resinosa* processed wood using hot sulfuric acid provided over 79% CCA removal [78]. Furthermore, effluents generated in acid decontamination were treated by precipitation with FeCl_3_ and NaOH or Ca(OH)_2_, as coagulant and alkalizing agents, respectively, displaying rates of removal over 98.5% [78].

Other techniques, such as electrodialysis and dialysis methods, were also applied to CCA-treated *Pinus pinaster* poles for removal of Cu, Cr and As from the maximum removal values (Cu, 84%; Cr, 87%; and As, 95%) were achieved under electrodialytic conditions generated for 14 days [79]. More recently, Jones et al. [80] recommended the separation and removal of metals in recycled construction and demolition wood, such as CCA, aiming to reduce adverse effects on the environment.

## 5. Conclusions

The present review emphasizes that, although the use of CCA has been banned for many years in several countries, and despite significant efforts being made to reduce its impact on the environment, its components still persist as documented in the literature. Previous studies have described that the bioavailability and bioaccessibility, and distribution of Cr, Cu and As in soils and aquatic ecosystems from CCA-treated wood and contaminated sites are element- and site-specific. Aged CCA-treated facilities (e.g., playgrounds, agricultural structures, zoological gardens) still pose risks for environmental, animal and human health due to leaching and accumulation of toxic elements. They should be specifically targeted in terms of maintenance or even removal. Individual components of CCA, namely As and Cr, pose severe human health problems, therefore, their removal from the environment should be promoted. In addition, the risk for both the environment and human health induced by Cu must be addressed through appropriate measures for its removal. Evidence suggests that arsenic, chromium and copper levels in surface soils, close to CCA-treated wood constructions, may surpass permitted levels, inducing concerns about both human and environmental health. The data discussed in this review show that it is of urgent need to act more efficiently in terms of the remediation of soils contaminated with CCA, and in terms of appropriate measures, such as hand washing after contact with CCA-wood and avoiding the contact of pets in these settings.
